# Concordance of three approaches for operationalizing outcome definitions for multidrug-resistant TB

**DOI:** 10.5588/ijtld.22.0324

**Published:** 2023-01-01

**Authors:** C. Zeng, C. D. Mitnick, C. Hewison, M. Bastard, P. Khan, K. J. Seung, M. L. Rich, S. Atwood, N. Melikyan, N. Morchiladze, N. Khachatryan, M. Khmyz, C. G. Restrepo, N. Salahuddin, E. Kazmi, A. A. Dahri, S. Ahmed, F. Varaine, S. C. Vilbrun, L. Oyewusi, A. Gelin, K. Tintaya, L. T. Yeraliyeva, S. Hamid, U. Khan, H. Huerga, M. F. Franke

**Affiliations:** 1 Department of Global Health and Social Medicine, Harvard Medical School, Boston, MA, USA; 2 Partners In Health (PIH), Boston, MA, USA; 3 Division of Global Health Equity, Brigham and Women’s Hospital, Boston, MA, USA; 4 Medical Department, Médecins Sans Frontières (MSF), Paris, France; 5 Field Epidemiology Department, Epicentre, Paris, France; 6 Interactive Research and Development Global, Singapore; 7 Clinical Research Department, Faculty of Infectious and Tropical Diseases, London School of Hygiene & Tropical Medicine, London, UK; 8 MSF, Sokhumi, Georgia; 9 MSF, Yerevan, Armenia; 10 MSF, Minsk, Belarus; 11 MSF, Yangon, Myanmar; 12 Indus Hospital & Health Network (IHHN), Karachi, Pakistan; 13 Center for Disease Control and Prevention, Directorate General Health Services, Sindh, Pakistan; 14 Interactive Research and Development, Karachi, Pakistan; 15 Haitian Group for the Study of Kaposi’s Sarcoma and Opportunistic Infections (GHESKIO), Port-au-Prince, Haiti; 16 PIH, Maseru, Lesotho; 17 Zanmi Lasante, Port-au-Prince, Haiti; 18 PIH/Socios En Salud Sucursal Peru, Lima, Peru; 19 National Scientific Center of Phthisiopulmonology of the Ministry of Health of the Republic of Kazakhstan, Kazakhstan; 20 Bishoftu General Hospital, Bishoftu, Ethiopia

**Keywords:** drug-resistant tuberculosis, rifampin-resistant tuberculosis, treatment outcome, definition

## Abstract

**BACKGROUND:**

The WHO provides standardized outcome definitions for rifampicin-resistant (RR) and multidrug-resistant (MDR) TB. However, operationalizing these definitions can be challenging in some clinical settings, and incorrect classification may generate bias in reporting and research. Outcomes calculated by algorithms can increase standardization and be adapted to suit the research question. We evaluated concordance between clinician-assigned treatment outcomes and outcomes calculated based on one of two standardized algorithms, one which identified failure at its earliest possible recurrence (i.e., failure-dominant algorithm), and one which calculated the outcome based on culture results at the end of treatment, regardless of early occurrence of failure (i.e., success-dominant algorithm).

**METHODS:**

Among 2,525 patients enrolled in the multi-country endTB observational study, we calculated the frequencies of concordance using cross-tabulations of clinician-assigned and algorithm-assigned outcomes. We summarized the common discrepancies.

**RESULTS:**

Treatment success calculated by algorithms had high concordance with treatment success assigned by clinicians (95.8 and 97.7% for failure-dominant and success-dominant algorithms, respectively). The frequency and pattern of the most common discrepancies varied by country.

**CONCLUSION:**

High concordance was found between clinician-assigned and algorithm-assigned outcomes. Heterogeneity in discrepancies across settings suggests that using algorithms to calculate outcomes may minimize bias.

## Abstract (fr)

**CONTEXTE:**

L’OMS offre des définitions standardisées des résultats pour la TB résistante à la rifampicine (RR) et multirésistante (MDR). Cependant, la mise en oeuvre de ces définitions peut s’avérer difficile dans certains contextes cliniques, et une classification incorrecte peut compromettre les rapports et la recherche. Les résultats calculés par des algorithmes peuvent accroître la standardisation et être adaptés à la question de recherche. Nous avons évalué la concordance entre les résultats du traitement attribués par le clinicien et les résultats calculés sur la base de l’un des deux algorithmes standardisés, l’un qui identifie l’échec à sa récurrence la plus précoce possible (c’est-à-dire l’algorithme à dominance d’échec), et l’autre qui calcule le résultat sur la base des résultats de la culture à la fin du traitement, indépendamment de la survenue précoce d’un échec (c’est-à-dire l’algorithme à dominance de succès).

**MÉTHODES:**

Parmi les 2 525 patients inscrits à l’étude observationnelle multipays endTB, nous avons calculé les fréquences de concordance en utilisant des tableaux croisés des résultats attribués par le clinicien et par l’algorithme. Nous avons résumé les divergences courantes.

**RÉSULTATS:**

Le succès du traitement calculé par les algorithmes présentait une concordance élevée avec le succès du traitement attribué par les cliniciens (95,8% et 97,7% pour les algorithmes à dominante échec et à dominante succès, respectivement). La fréquence et le modèle des divergences les plus courantes variaient selon les pays.

**CONCLUSION:**

Une grande concordance a été constatée entre les résultats attribués par les cliniciens et ceux attribués par les algorithmes. L’hétérogénéité des divergences entre les contextes suggère que l’utilisation d’algorithmes pour calculer les résultats peut minimiser les irrégularités.

Evaluating end-of-treatment (EOT) outcomes for rifampicin-resistant (RR) and multidrug-resistant (MDR) TB is important for clinical care and programmatic monitoring. To facilitate the outcome evaluation for RR/MDR-TB, the WHO provides standardized outcome definitions.^1^

Observational research plays an important role in generating evidence to guide RR/MDR-TB treatment recommendations. Standardized application of outcome definitions when analyzing RR/MDR-TB cohorts is one step, among many, that investigators may take to generate valid, comparable results across studies.^2^–^5^ In practice, however, applying standardized definitions, which include the consideration of both quantity and timing of culture results, may be challenging.^1^,^6^ In clinical settings, consistent outcome assignment may be complicated by differences in treatment duration; availability of microbiological results (relative to the timing of cessation of treatment); availability and use of clinical and radiographic data; adherence to treatment; and information on reasons for treatment change.^6^ To enhance standardization, algorithms can be used to calculate outcomes based on pre-specified criteria. Algorithms can also be adapted for different purposes, such as evaluating the effectiveness of an initial regimen without considering the effects of subsequent treatment adjustments or the effectiveness of overall treatment strategies after considering such effects. However, using these algorithms involves numerous decision points guided by careful consideration of the research question.

In this study, we applied WHO outcome definitions to a cohort of patients treated for RR/MDR-TB. We programmed two algorithms, each for a distinct research purpose, and compared algorithm-assigned outcomes to clinician-assigned outcomes, with the overall goal of understanding the extent to which different operationalizations of the same definitions may lead to diverging outcome assignment and potential bias in observational RR/MDR-TB treatment cohorts.

## METHODS

### Data resource, study design, and participants

Data were derived from the endTB observational study, a prospective cohort of patients treated for RR/MDR-TB with an individualized, longer regimen containing bedaquiline- and/or delamanid, composed according to WHO and local guidance, in one of 17 countries.^7^ A total of 2,789 patients were recruited and enrolled between April 2015 and September 2018. Routine clinical and laboratory data were entered into an electronic medical record. For this analysis, we excluded patients treated in the Democratic People’s Republic of Korea (*n* =155, the only participating country that a priori implemented shortened regimens for RR/MDR-TB), and those for whom there was no recorded clinician-assigned outcome (*n* = 82) or an outcome of not evaluated, transferred, or “treatment adapted” (*n* = 27).

### WHO outcome definitions

Outcome assignments were based on the 2013 WHO definitions in place during the study period.^1^ Under these definitions, an outcome of cure was assigned if patients completed treatment with three or more consecutive negative cultures taken at least 30 days apart after the intensive phase, or after 8 months if there was no intensive phase. Patients whose treatment outcome did not meet the definition of cure but in whom there was no evidence of treatment failure are assigned an outcome of treatment completed. Treatment failure was defined as treatment termination or need for a permanent regimen change of two or more anti-TB drugs due to any of the following reasons: lack of culture conversion by the end of the intensive phase; bacteriological reversion in the continuation phase; detection of acquired resistance to fluoroquinolones or second-line injectable drugs; or adverse drug reactions. Death was defined as death from any cause during treatment. Loss to follow-up (LTFU) was said to have occurred after a treatment interruption of 2 consecutive months.

### Operationalization of WHO outcome definitions Clinician-assigned outcomes

As part of routine care, clinicians assigned a treatment outcome to each patient based on available laboratory results and clinical information, in accordance with WHO outcomes.^1^

### Algorithm-assigned outcomes

We created two algorithms based on WHO outcome definitions. The first was designed to establish failure at its earliest possible occurrence (henceforth, the “failure-dominant algorithm”). The second assigned outcomes based on culture results available at the end of treatment, regardless of whether the patient’s experience initially met the definition for early treatment failure (henceforth, the “success-dominant algorithm”). Outcomes generated under the success-dominant algorithm reflected treatment response to the initial regimen and any subsequent regimen changes (i.e., those occurring in response to early treatment failure). For example, a patient who underwent early bacteriological reversion from culture-negative to positive, but whose experience ultimately met the definition for cure at the end of treatment, would be assigned an outcome of failure under the failure-dominant algorithm and an outcome of cure under the success-dominant algorithm.

Outcomes under each algorithm were calculated as follows. The failure-dominant algorithm (Supplementary Figure S1) identified the earliest date of treatment failure after 8 months of treatment (as proxy for the intensive phase). Patients who did not experience treatment failure and were not assigned an outcome of death remained eligible for an outcome of success, which was calculated as in the success-dominant algorithm. In the success-dominant algorithm (Supplementary Figure S2), patients were assigned an outcome at the end of treatment based on the available culture results. Longer regimens used were intended to last 18–20 months; in some instances, however, clinicians stopped treatment before 18 months. To reflect the intent that all included treatments were longer regimens, we imposed a minimum treatment duration of 15 months for an outcome assignment of cure or treatment completion.^8^ Patients who completed 15 months of treatment were assigned an outcome based on WHO outcome definitions, regardless of whether they became LTFU after this period. Outcome of death was assigned when it occurred. Patients who became LTFU before completing 15 months of treatment were assigned LTFU. Patients who were treated for less than 15 months without indication of LTFU or death were assigned an outcome as follows: 1) treatment failure if patients had at least two cultures after 8 months of treatment and at least one of the following was true: more than one of the last three cultures were positive or the final culture was positive; 2) “<15 months, favorable” if patients had a maximum of one positive culture and the final culture was negative; 3) “<15 months, unfavorable” if there were less than two cultures after 8 months of treatment but there was no indication of “<15 months, favorable”.

### Treatment success

Outcomes of cured, treatment completed, and “<15 months, favorable” were classified as treatment success. Outcomes of died, failed, LTFU, and “<15 months, unfavorable” were classified as no success.

### Statistical analysis

We calculated the frequencies of patients with each outcome across the three approaches to operationalizing WHO outcome definitions. We also calculated the frequencies of concordance between algorithm-assigned and clinician-assigned outcomes for each individual outcome and for the broader dichotomous category of treatment success versus no success. To evaluate the overall concordance between clinician-assigned and algorithm-assigned outcomes, we calculated the simple κ coefficient and 95% confidence intervals (CIs) for the dichotomous category of treatment success. We summarized the common discrepancies.

### Research ethics

The endTB observational study protocol was approved by all study countries (Armenia, Bangladesh, Belarus, Ethiopia, Georgia, Haiti, Indonesia, Kazakhstan, Kenya, Kyrgyzstan, Lesotho, Myanmar, Pakistan, Peru, South Africa, Vietnam) and central ethics review committees for each consortium partner. Patients provided written informed consent for inclusion in the observational cohort.

## RESULTS

### Overview

After exclusions, 2,525 patients with RR/MDR-TB were included for analyses (Supplementary Figure S3). Overall, the frequencies of treatment success were 78.3% (1,978/2,525; 95% CI 72.7–83.1), 77.4% (1,955/2,525; 95% CI 71.8–82.3), 80.0% (2,021/2,525; 95% CI 74.5–84.6) using clinician-assigned, failure-dominant and success-dominant outcome definitions, respectively (Table 1).

**Table 1:** Frequencies and proportions of treatment outcomes across the three operationalization approaches (N = 2,525)

Outcomes	Clinician-assigned outcomes *n* (%)	Algorithm-defined failure-dominant *n* (%)	Algorithm-defined success-dominant *n* (%)
Success, *n* (%) (95% CI)*	1,978 (78.3) (72.7–83.1)	1,955 (77.4) (71.8–82.3)	2,021 (80.0) (74.5–84.6)
Cured	1,852 (73.4)	1,835 (72.7)	1,891 (74.9)
Treatment completed	126 (5.0)	45 (1.8)	53 (2.1)
<15 months, favorable	—	75 (3.0)	77 (3.1)
No success, *n* (%) (95% CI)^†^	547 (21.7) (20.1–23.3)	570 (22.6) (21.0–24.3)	504 (20.0) (18.4–21.6)
Died	232 (9.2)	224 (8.9)	232 (9.2)
Treatment failed	109 (4.3)	145 (5.7)	69 (2.7)
LTFU	206 (8.2)	181 (7.2)	183 (7.3)
<15 months, unfavorable	—	20 (0.8)	20 (0.8)

* Outcomes of cured, completed, and “<15 months, favorable”.

† Outcomes of died, failed, LTFU, and “<15 months, unfavorable”.

CI = confidence interval; LTFU = lost to follow-up.

### Concordance between clinician-assigned and algorithm-assigned outcomes

The frequency of concordance between clinician-assigned outcomes and algorithm-assigned failure-dominant outcomes for the dichotomous category of treatment success was 95.8% (2,418/2,525) and for individual outcomes was 87.2% (2,202/2,525) (Table 2). The κ coefficient for treatment success was 0.88 (95% CI 0.85–0.90). For the algorithm-assigned success-dominant outcomes, the frequency of concordance for treatment success was 97.7% (2,466/2,525) and for individual outcomes was 89.4% (2,256/2,525) (Table 2). The κ coefficient for treatment success was 0.93 (95% CI 0.91–0.95).

**Table 2:** Concordance between clinician-assigned outcomes and algorithm-assigned outcomes, all study countries (N = 2,525)

Clinician-assigned outcomes	Algorithm-assigned outcomes								

	Cured *n* (%)	Treatment completed *n* (%)	Died *n* (%)	Treatment failed *n* (%)	LTFU *n* (%)	< 15 months, favorable *n* (%)	<15 months, unfavorable *n* (%)	Success* *n* (%)	No success^†^ *n* (%)
Concordance between clinician-assigned outcomes and failure-dominant, algorithm-assigned outcomes									
Cured	1,718 (68.0)^‡^	28 (8.7)		58 (18.0)		48 (14.9)		—	—
Treatment completed	96 (29.7)	13 (0.5)^‡^		7 (2.2)		10 (3.1)		—	—
Died	1 (0.3)		224 (8.9)^‡^	7 (2.2)				—	—
Treatment failed	4 (1.2)	2 (0.6)		66 (2.6)^‡^		17 (5.3)	20 (6.2)	—	—
LTFU	16 (5.0)	2 (0.6)		7 (2.2)	181 (7.2)^‡^			—	—
Success*	—	—	—	—	—	—	—	1,913 (75.8)^‡^	65 (60.7)
No success^†^	—	—	—	—	—	—	—	42 (39.3)	505 (20.0)^‡^
Concordance between clinician-assigned outcomes and success-dominant, algorithm-assigned outcomes									
Cured	1,767 (70.0)^‡^	29 (10.8)		7 (2.6)		49 (18.2)		—	—
Treatment completed	98 (36.4)	17 (0.7)^‡^		1 (0.3)		10 (3.7)		—	—
Died			232 (9.2)^‡^					—	—
Treatment failed	9 (3.4)	5 (1.9)		57 (2.3)^‡^		18 (6.7)	20 (7.4)	—	—
LTFU	17 (6.3)	2 (0.7)		4 (1.5)	183 (7.2)^‡^			—	—
Success*	—	—	—	—	—	—	—	1,970 (78.0)^‡^	8 (13.6)
No success^†^	—	—	—	—	—	—	—	51 (86.4)	496 (19.7)^‡^

* Cured, completed, and “<15 months, favorable”.

† Died, failed, LTFU, and “<15 months, unfavorable”.

‡ Percentages are calculated from the overall population (*N* = 2,525). Percentages are calculated among discordant outcomes in the remaining cells.

LTFU = lost to follow-up.

In a sensitivity analysis excluding patients with outcomes of “<15 months, unfavorable” and “<15 months, favorable”, the frequencies of concordance for treatment success remained high, at 96.3% (2,340/2,430), for the failure-dominant algorithm, and 98.3% (2,387/2,428) for the success-dominant algorithm.

### Discrepancies between clinician-assigned and algorithm-assigned outcomes

Most discrepancies in individual outcomes (216/323 [66.9%] for the failure-dominant algorithm and 210/269 [78.1%] for the success-dominant algorithm) did not affect whether the outcome was classified as successful. Of the discrepancies identified, there were two common patterns: one involved a clinician-assigned outcome of treatment completed which was classified as cured by the algorithms; another pattern involved a clinician-assigned outcome of cured which was classified as “<15 months, favorable” in algorithm-assigned outcomes.

**Figure 1:**
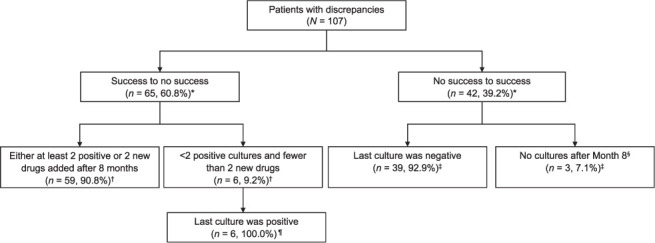
Discrepancies between clinician-assigned outcomes and failure-dominant-assigned outcomes. *Denominator = total number of patients with discrepancies (n= 107). ^†^Denominator = number of patients with change from success to no success (n=65). ^‡^Denominator = number of patients with change from no success to success (n = 42). ^§^Patients treated <15 months who had no culture results; assigned an outcome of “<15 months, favorable”. ^¶^Denominator = number of patients who had <2 positive cultures and two new drugs were added after 8 months (n = 6).

A total of 107/323 (33.1%) and 59/269 (21.9%) discrepancies between clinician-assigned outcomes and the failure-dominant and success-dominant algorithms, respectively, affected whether the outcome was classified as successful. Among the discrepancies between clinician-assigned outcomes and those calculated with the failure-dominant algorithm, 65 (60.8%) patients were assigned an outcome indicative of treatment success by the clinician but were classified as no success by the failure-dominant algorithm (Figure 1). Most of these discrepancies (*n* = 59, 90.8%) occurred when a clinician assigned an outcome of cured or completed in the presence of at least two positive cultures or after the addition of two new drugs added to the initial regimens by the end of 8 months of treatment. The remaining 42 of 107 patients (39.2%) were assigned an outcome of unsuccessful treatment by the clinician but were classified as an outcome of successful treatment by the algorithm.

**Figure 2:**
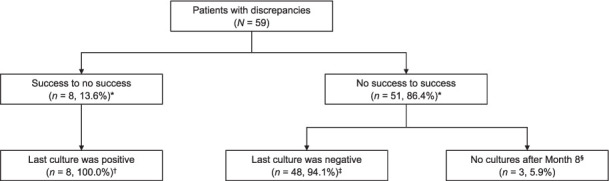
Discrepancies between clinician-assigned outcomes and success-dominant-assigned outcomes. *Denominator = total number of patients with discrepancies (n = 59). ^†^Denominator = number of patients with change from success to no success (n = 8). ^‡^Denominator = number of patients with change from no success to success (n = 51). ^§^Patients were treated <15 months and had no culture results; assigned an outcome of “<15 months, favorable”.

For discrepancies between clinician-assigned outcomes and those calculated with the success-dominant algorithm (Figure 2), the prevailing pattern, which accounted for 86.4% (51/59) of the total discrepancies, was one in which clinicians assigned an outcome of unsuccessful treatment while the algorithm(s) assigned an outcome of successful treatment. Of 51 discrepancies, 32 patients had a clinician-assigned outcome of failure and 19 of LTFU. Most (*n* = 48, 94.1%) of these outcomes were classified as treatment success by the algorithm because the final cultures were negative.

### Heterogeneity in the frequency and type of discrepancies by country

Country-specific sample sizes ranged from 5 to 671 (Table 3). Across countries, the frequency of discrepancies between clinician-assigned outcomes and those from each algorithm yielded heterogeneous results. For example, in Country 1, discrepancies typically involved a clinician-assigned outcome of unsuccessful treatment and an algorithm-assigned outcome of successful treatment (8/10 [80%] and 9/11 [81.8%] for failure-dominant and success-dominant algorithms, respectively). In Country 8, discrepancies between clinician-assigned outcomes and those from the failure-dominant algorithm tended to go both ways (i.e., 19/31 [61.3%] of discrepancies involved a clinician-assigned outcome of treatment success and algorithm-assigned outcome of no success, while 12/31 [38.7%] of discrepancies involved a clinician-assigned outcome indicative of no treatment success and algorithm-assigned outcome of success). Comparing clinician-assigned outcomes to those derived from the success-dominant algorithm in Country 8 revealed 17 discrepancies, all of which were clinician-assigned outcomes of no treatment success and algorithm-assigned outcomes of success.

**Table 3:** Discordance between clinician-assigned and algorithm-assigned outcomes by study country (N = 2,525)

Country	Treatment success in clinician-assigned outcomes *n* (%)	Treatment success in algorithm-assigned outcomes *n* (%)	Difference *n* (%)	Discrepancies *n* (%)	Success to no success *n* (%)	No success to success *n* (%)
Clinician-assigned outcomes compared to algorithm-assigned outcomes (failure-dominant algorithm)						
Country 1 (*n* = 105)	48 (45.7)	54 (51.4)	6 (5.7)	10 (9.5)	2 (1.9)	8 (7.6)
Country 2 (*n* = 279)	239 (85.7)	238 (85.3)	1 (0.4)	9 (3.2)	5 (1.8)	4 (1.4)
Country 3 (*n* = 84)	64 (76.2)	64 (76.2)	0 (0.0)	4 (4.8)	2 (2.4)	2 (2.4)
Country 4 (*n* = 64)	49 (76.6)	48 (75.0)	1 (1.6)	3 (4.7)	2 (3.1)	1 (1.6)
Country 5 (*n* = 288)	228 (79.2)	223 (77.4)	5 (1.7)	17 (5.9)	11 (3.8)	6 (2.1)
Country 6 (*n* = 13)	10 (76.9)	10 (76.9)	0 (0.0)	0 (0.0)	0 (0.0)	0 (0.0)
Country 7 (*n* = 72)	36 (50.0)	36 (50.0)	0 (0.0)	4 (5.6)	2 (2.8)	2 (2.8)
Country 8 (*n* = 671)	589 (87.8)	582 (86.7)	7 (1.0)	31 (4.6)	19 (2.8)	12 (1.8)
Country 9 (*n* = 5)	3 (60.0)	4 (80.0)	1 (20.0)	1 (20.0)	0 (0.0)	1 (20.0)
Country 10 (*n* = 13)	5 (38.5)	6 (46.2)	1 (7.7)	1 (7.7)	0 (0.0)	1 (7.7)
Country 11 (*n* = 247)	182 (73.7)	174 (70.5)	8 (3.2)	8 (3.2)	8 (3.2)	0 (0.0)
Country 12 (*n* = 38)	21 (55.3)	22 (57.9)	1 (2.6)	1 (2.6)	0 (0.0)	1 (2.6)
Country 13 (*n* = 302)	216 (71.5)	211 (69.9)	5 (1.7)	9 (3.0)	7 (2.3)	2 (0.7)
Country 14 (*n* = 266)	228 (85.7)	228 (85.7)	0 (0.0)	2 (0.8)	1 (0.4)	1 (0.4)
Country 15 (*n* = 46)	33 (71.7)	31 (67.4)	2 (4.3)	4 (8.7)	3 (6.5)	1 (2.2)
Country 16 (*n* = 32)	27 (84.4)	24 (75.0)	3 (9.4)	3 (9.4)	3 (9.4)	0 (0.0)
Clinician-assigned outcomes compared to algorithm-assigned outcomes (success-dominant algorithm)						
Country 1 (*n* = 105)	48 (45.7)	55 (52.4)	7 (6.7)	11 (10.5)	2 (1.9)	9 (8.6)
Country 2 (*n* = 279)	239 (85.7)	242 (86.7)	3 (1.1)	3 (1.1)	0 (0.0)	3 (1.1)
Country 3 (*n* = 84)	64 (76.2)	67 (79.8)	3 (3.6)	3 (3.6)	0 (0.0)	3 (3.6)
Country 4 (*n* = 64)	49 (76.6)	49 (76.6)	0 (0.0)	2 (3.1)	1 (1.6)	1 (1.6)
Country 5 (*n* = 288)	228 (79.2)	234 (81.3)	6 (2.1)	6 (2.1)	0 (0.0)	6 (2.1)
Country 6 (*n* = 13)	10 (76.9)	10 (76.9)	0 (0.0)	0 (0.0)	0 (0.0)	0 (0.0)
Country 7 (*n* = 72)	36 (50.0)	38 (52.8)	2 (2.8)	2 (2.8)	0 (0.0)	2 (2.8)
Country 8 (*n* = 671)	589 (87.8)	606 (90.3)	17 (2.5)	17 (2.5)	0 (0.0)	17 (2.5)
Country 9 (*n* = 5)	3 (60.0)	4 (80.0)	1 (20.0)	1 (20.0)	0 (0.0)	1 (20.0)
Country 10 (*n* = 13)	5 (38.5)	7 (53.9)	2 (15.4)	2 (15.4)	0 (0.0)	2 (15.4)
Country 11 (*n* = 247)	182 (73.7)	181 (73.2)	1 (0.4)	1 (0.4)	1 (0.4)	0 (0.0)
Country 12 (*n* = 38)	21 (55.3)	22 (57.9)	1 (2.6)	1 (2.6)	0 (0.0)	1 (2.6)
Country 13 (*n* = 302)	216 (71.5)	216 (71.5)	0 (0.0)	4 (1.3)	2 (0.7)	2 (0.7)
Country 14 (*n* = 266)	228 (85.7)	231 (86.8)	3 (1.1)	3 (1.1)	0 (0.0)	3 (1.1)
Country 15 (*n* = 46)	33 (71.7)	34 (73.9)	1 (2.2)	1 (2.2)	0 (0.0)	1 (2.2)
Country 16 (*n* = 32)	27 (84.4)	25 (78.1)	2 (6.3)	2 (6.3)	2 (6.3)	0 (0.0)

## DISCUSSION

Outcomes derived using each of two algorithms had high concordance with clinician-assigned outcomes, suggesting that all three approaches generally yielded outcomes that reflect WHO outcome definitions. Although relatively rare, we identified patterns of discrepancies between clinician-assigned and algorithm-assigned outcomes that varied in frequency across settings. For example, the percentage of discrepancies between clinician-assigned and failure-dominant algorithm-assigned outcomes ranged from 0 to 20%. While small sample sizes in some countries likely contributed to variability, heterogeneity could also be due to differences in training, experience, and the flexibility with which outcome definitions are applied by clinicians, all of which may differ by setting, leading to varying degrees of bias.^9^ Standardized algorithms that calculate outcomes based on available data may reduce human error and heterogeneity in outcome assignment, and thereby reduce bias.

We also found that types of discordancy, such as treatment success by clinicians to no success by algorithms (or vice versa), differed across settings. For example, in some countries, discrepancies occurred in only one direction, while in others, they occurred in both directions. Although the latter may not necessarily bias the absolute proportion, it will cause bias in risk factor and comparative effectiveness analysis or individual patient data meta-analysis if the approaches used for outcome assignment differ by setting. Regimens are also often clustered by setting based on the national TB program guidelines for regimen composition; if some countries or clinicians apply more or less stringent criteria (i.e., the minimum duration of treatment used to define cure) for outcome classification, bias may occur and preclude correct conclusions regarding relative treatment effectiveness. Operationalizing WHO outcome definitions using a consistent approach across settings and reporting the approach used for outcome assignment could reduce such bias.

The approach used for outcome assignment should align with the intended purpose.^6^,^10^–^12^ Calculated outcomes may reduce heterogeneity across time and place while allowing flexibility to contend with changing outcome definitions and address specific research objectives.^10^–^12^ For example, the failure-dominant algorithm may be most appropriate for evaluating the effectiveness of an initial regimen because treatment failure is defined as soon as it is identified at the end of 8 months. In this way, the failure-dominant algorithm evaluates treatment response to the initial regimen, but not the combined response of the initial regimen plus subsequent regimen changes made due to treatment failure. Alternatively, the success-dominant algorithm designates an outcome at the end of treatment, regardless of evidence of early treatment failure and subsequent regimen changes; this definition may be most appropriate for evaluating the effectiveness of overall treatment and management strategies, as opposed to the effectiveness of an individual regimen. The TRUNCATE-TB trial provides an informative, illustrative example of assessing a “strategy”. Study patients who experience relapse after the 8-week shortened regimen will be treated with a standard 24-week regimen subsequently.^13^ The primary efficacy endpoint, assessed at 96 weeks after randomization, represents a combined strategy for treatment and management of any subsequent relapse. Our success- dominant algorithm aligns with this approach in that it considers the overall treatment experience, including the response to the initial regimen and the management of early treatment failure.

Although algorithms could improve outcome classification by removing bias and variability, clinician-assigned outcomes may sometimes most accurately capture the intent of definitions (e.g., if there were laboratory errors that contributed to a final positive culture in the absence of clinical symptoms). This scenario represents an inherent limit to algorithms; these rely on available data and could be biased if factors considered by clinicians are not available to the analysis team. A second example would be if a culture result was available in clinical chart but not entered into the database. Likewise, algorithms are less informed than clinician-assigned outcomes if culture results are missing. Missingness of culture results could be due to reagent stock-outs, limited laboratory capacity, or a patient’s inability to produce an adequate sputum sample (especially during the later stages of treatment).^6^ For patients who were unable to produce sputum samples, missing cultures could be considered negative.^12^ Recording the reasons for missing sputum samples or culture results, and reasons for necessary deviations from standardized definitions may facilitate further honing of WHO outcome definitions and associated algorithms for calculated outcomes.

Evidence from this study supports the use of algorithms to calculate EOT outcomes for RR/MDR-TB research cohorts. Doing so may be particularly important in multisite studies, or those with follow-up periods encompassing different outcome definitions, in order to reduce heterogeneity across countries and among clinicians and to minimize bias.
